# Ecological impact of a secondary bacterial symbiont on the clones of *Sitobion avenae* (Fabricius) (Hemiptera: Aphididae)

**DOI:** 10.1038/srep40754

**Published:** 2017-01-17

**Authors:** Chen Luo, Kun Luo, Linqin Meng, Bin Wan, Huiyan Zhao, Zuqing Hu

**Affiliations:** 1 State Key Laboratory of Crop Stress Biology for Arid Areas, College of Plant Protection, Northwest A&F University, Yangling, Shaanxi Province 712100, China; 2 INRA (French National Institute for Agricultural Research), Univ. Nice Sophia Antipolis, CNRS, UMR 1355-7254 Institut Sophia Agrobiotech, 06903, Sophia Antipolis, France

## Abstract

Many insects harbor heritable endosymbionts, whether obligatory or facultative, and the role of facultative endosymbionts in shaping the phenotype of these species has become increasingly important. However, little is known about whether micro-injected endosymbionts can have any effects on aphid clones, which was measured using various ecological parameters. We examined the effects between symbiotic treatments and the vital life history traits generated by *Regiella insecticola* on the life table parameters of *Sitobion avenae.* The results showed that *R. insecticola* can decrease the intrinsic rate of increase (*r*), the finite rate of increase (*λ*) and birth rate and can increase the mean generation times (*T*) of *S. avenae* clones, suggesting that *R. insecticola* may decelerate the normal development of the hosts. No significant differences of these parameters were observed between the examined *Sitobion avenae* clones, and the symbiont treatment by genotype interaction affected only the net reproduction rate *R*_*0*_, pre-adult duration and total longevity but not the other parameters. Additionally, a population projection showed that *R. insecticola* decelerated the growth of the *S. avenae* clones. The evocable effects of *R. insecticola* on the *S. avenae* clones may have significant ramifications for the control of *S. avenae* populations under field/natural conditions.

Many insect species harbor endosymbionts, which are indispensable to their survival and common reproduction. Because the hosts and their endosymbionts share a common fate, endosymbionts can often profoundly affect the ecology and evolution of their hosts. Among them, the endosymbionts in aphids are ideal targets for studying their role in ecological fitness^1^. In nature, almost all aphid species possess the primary endosymbiont *Buchnera aphidicola*, which belongs to the class *γ*-proteobacterium. *B. aphidicola* synthesizes essential amino acids and other nutrients that are ingested in very small quantities from restricted diets, such as plant phloem, for their host aphids^2^,^3^,^4^. Moreover, aphids can also be infected with one or more non-essential secondary endosymbionts belonging to distinct bacterial lineages^1^. For example, *Regiella insecticola, Hamiltonella defensa* and *Serratia symbiotica* belong to the class *γ*-proteobacterium, and *Rickettsia* and *Spiroplasma* belong to the class *α*-proteobacterium^5^,^6^,^7^,^8^,^9^,^10^,^11^,^12^,^13^.

Secondary endosymbionts do not present in all aphid individuals, suggesting they are not generally required for the survival and reproduction of aphids. However, endosymbionts can significantly affect host aphid ecological fitness and behavior. In the past few decades, secondary endosymbionts have been found to confer benefits on the pea aphid *Acyrthosiphon pisum* (Harris), including resistance to heat shock (*S. symbiotica*^14^), protection against natural enemies (*H. defensa* and *S. symbiotica*^15^, *H. defensa*^16^, *R. insecticola*^17^, *Spiroplasma*^18^ and *Rickettsiella*^19^), adjustment to reproduction (*Spiroplasma*^20^), host utilization (*R. insecticola*^21^), wing and sexual induction (*R. insecticola*^22^) and body color (*Rickettsiella*^23^). To date, there is evidence that secondary endosymbionts play major roles in protecting host aphids against natural enemies^17^,^24^, and their effects on host aphid population dynamics^25^ can affect the communities involved^26^. To examine the dynamic population parameters affected by endosymbionts, an appropriate study of the life history of aphids is essential. Wang *et al*.^27^ reported the life history of *R. insecticola* in *S. avenae* using antibiotics to selectively cure infected lines (the elimination of secondary symbionts using 100 μg ml^−1^ ampicillin, 50 μg ml^−1^ cefotaxime and 50 μg ml^−1^ gentomicin). However, a key component of this knowledge is the life table. The life table analysis is the most reliable method for evaluating the survival and reproduction of a population^28^, and some vital ecological parameters are derived from a life table analysis. For instance, the intrinsic rate of increase (*r*) represents the reproductive potential of a population in relation to its capacity to increase^29^; the finite rate of increase (λ) was defined as the rate of increase per individual per unit time; and the net reproductive rate (*R*_*0*_) has been used to summarize the mean number of offspring that an individual can produce during its lifetime, with the mean generation time (*T*) defined as the period that a population needs to increase its size *R*_*0*_-fold^30^. At the same time, the inoculation (by injection or feeding) and elimination of secondary symbionts are complementary methods. It is often best to use both methods to demonstrate an effect of these bacteria, and adding and removing bacteria can present the predicted phenotypic effects. However, elimination of secondary symbionts with antibiotics may generate the concurrent harm to the primary symbiont, *Buchnera*, on which aphid performance and fecundity depends^15^. Therefore, for a more exact understanding of the impacts of *R. insecticola* on hosts, it is necessary to obtain *S. avenae* clones with *R. insecticola* inoculated via micro-injection, which would have the same genetic background as symbiont-free aphids. Additionally, different aphid species or different genetic backgrounds within one species in the field can harbor various endosymbionts, and it is not clear whether the genotypes of the hosts could impact the ecological effects of endosymbionts. Therefore, the study of the interactive effects between micro-injected endosymbionts and the genotypes of the hosts and the ecological effects of endosymbionts on the aphid population is warranted.

The objectives of the study are to investigate the ecological effects of *R. insecticola* on different *S. avenae* clones based on the life table data. The results that we present here might provide new insight for the future systematic study of endosymbionts and its ramifications in suppressing *S. avenae* populations.

## Results

### The effects of *R. insecticola* on population parameters in different aphid genotypes (clones)

A two-way nested analysis of variance was used to analyze the effect of endosymbionts on the population parameters in the different genotypes of aphid host (Table 1). *R. insecticola* significantly impacted the population parameters (i.e., *P* < 0.001 for the intrinsic rate of increase *r*, mean generation times *T*, finite rate of increase *λ* and net reproductive rate *R*_*0*_). Clone nested in ‘*R. insecticola*’ had no impact on *r (P* = 0.091), *T (P* = 0.236) and *λ (P* = 0.090), but significantly impacted *R*_*0*_(*P* = 0.001).

The population parameters of these original aphid clones (controls) were compared to those corresponding to infected clones (treatments) (Table 2). Body fluids from the Yangpingguan clones 1 and 2 were transferred to the Linyi clone and the Neixiang clone, respectively, resulting in the creation of four new clones (Linyi-1 and Neixiang-1 from Yangpingguan clone 1 donor, Linyi-2 and Neixiang-2 from Yangpingguan clone 2 donor). The four clones presented significantly lower values (*F* = 27.418, *P* < 0.001 and *F* = 28.216, *P* < 0.001 for *r* and *λ*, respectively) than the controls Linyi-NA (Linyi clone) and Neixiang-NA (Neixiang clone). There were no differences between the treatments (Linyi-1, Linyi-2, Neixiang-1 and Neixiang-2) (*P* = 0.191 and *P* = 0.194 for *r* and *λ*, respectively). However, the mean generation times (*T*) of treatments Linyi-1, Linyi-2, Neixiang-1 and Neixiang-2 were significantly longer (*F* = 19.553, *P* < 0.001) than that of the controls Linyi-NA and Neixiang-NA, and no differences were found between the treatments (Linyi-1, Linyi-2, Neixiang-1 and Neixiang-2) (*P* = 0.119).

### The effects of *R. insecticola* on the development and longevity of different *S. avenae* genotypes

A two-way nested analysis of variance was used to analyze the effect of endosymbionts on fitness traits in the different genotypes of the aphid host (Table 3). *R. insecticola* clearly affected the birth rate (*P* < 0.001), pre-adult duration (*P* < 0.001) and total longevity (*P* = 0.001). Clone nested in ‘*R. insecticola*’ had no impact on birth rate (*P* = 0.091) but had a significant impact on pre-adult duration and total longevity (*P* = 0.018 and *P* = 0.004, respectively).

Fitness traits (i.e., birth rates, pre-adult duration, and total longevity) of the controls clones were compared to those corresponding to infected clones (treatments) (Table 4). *R. insecticola* had a remarkable effect on birth rate, and birth rates in the treatments Linyi-1, Linyi-2, Neixiang-1 and Neixiang-2 were significantly lower (*F* = 30.404, *P* < 0.001) than that in the controls Linyi-NA and Neixiang-NA. No differences in birth rate were found between the treatments (Linyi-1, Linyi-2, Neixiang-1 and Neixiang-2) (*P* = 0.163). The pre-adult duration of the treatments Linyi-1, Linyi-2, Neixiang-1 and Neixiang-2 (8.32 ± 0.062, 7.51 ± 0.156, 7.80 ± 0.102 and 7.97 ± 0.259, respectively) were clearly longer than that of the controls Linyi-NA (7.03 ± 0.126) and Neixiang-NA (6.62 ± 0.950). In addition, the total longevity of the treatments Linyi-1, Linyi-2, Neixiang-1 and Neixiang-2 (27.34 ± 0.484, 27.62 ± 0.624, 26.31 ± 0.791 and 25.41 ± 0.423, respectively) were clearly higher than that of the control Linyi-NA (22.29 ± 0.756).

### Population projection

**Figure 1:**
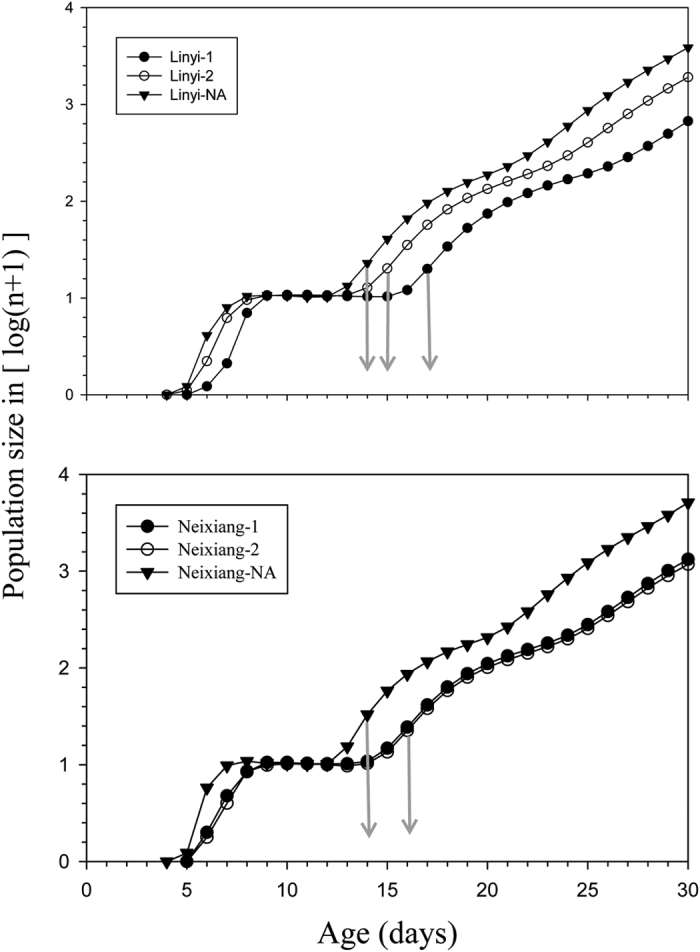
Simulated adult population growth of six *S. avenae* clones (Linyi-1, Linyi-2, Neixiang-1, Neixiang-2, Linyi-NA and Neixiang-NA) for 30 days. Linyi genotypes with *R. insecticola* (Linyi-1 and Linyi-2) donated by Yangpingguan 1 and Yangpingguan 2, respectively; Neixiang genotypes with *R. insecticola* (Neixiang-1 and Neixiang-2) donated by Yangpingguan 1 and Yangpingguan 2, respectively. Linyi-NA represents Linyi genotype without *R. insecticola* and Neixiang-NA represents Neixiang genotype without *R. insecticola*. The arrow heads point to the times in which the populations entered sustained population growth (outbreak times).

The simulated population growth and stage structure of the adult stages for 30 days, beginning with an initial population of 10 pairs of 1^st^ instar nymphs, are shown in Fig. 1. For the adults, after an early stable phase, the times in which the populations entered sustained population growth (outbreak times) for the treatments Linyi-1, Linyi-2, Neixiang-1 and Neixiang-2 occurred on the 17^th^, 15^th^, 16^th^ and 16^th^ day, respectively, and the corresponding controls Linyi-NA and Neixiang-NA both occurred on the 14^th^ day. This showed that the outbreak times of the treatments were delayed compared to those of the controls, and it suggested that *R. insecticola* might postpone the timing of the adult populations in the studied *S. avenae* clones.

## Discussion

The diverse infections and functions of secondary endosymbionts of aphids (e.g., *A. pisum, Aphis craccivora* and *Myzus persicae*) have been extensively studied in recent decades^5^,^6^,^7^,^8^,^9^,^18^,^31^,^32^,^33^,^34^,^35^,^36^. In addition, it has become clear that secondary endosymbionts have major effects on the ecology of the pea aphid and arthropods in general. For example, *H. defensa* can protect *A. pisum* from its natural enemies^1^,^16^,^18^,^24^. However, Łukasik *et al*.^31^ reported that *H. defensa* has little effect on the resistance to the natural enemies in *S. avenae*, indicating that secondary endosymbionts might, to some extent, have different effects on related species. Therefore, it is necessary to study the effects of the secondary symbionts on different aphid clones using the life table.

A linear two-way nested analysis of variance (ANOVA) showed that the treatment by genotype interaction did not affect aphid fitness (e.g., *r, T, λ* and birth rate), though the interaction did affect the net reproductive rate (*R*_0_), pre-adult duration and total longevity. For a comprehensive understanding of the treatment by genotype interaction, more studies are needed.

Understanding the entire life history plays a significant role in evaluating the population parameters that are influenced by secondary endosymbionts, and the key component of this analysis is the life table, which can provide a comprehensive description of the development, survival, and fecundity of a population^28^. Wang *et al*.^27^ obtained the “curing of *R. insecticola* infected lines” through an oral administration of antibiotics, and they studied the change in the developmental time of 1^st^ to 4^th^ instar nymphs (DT1-DT4), the total developmental time of nymphs (DT5), and 10 d fecundity. However, their research did not focus on the population parameters. In life table research, the population parameters represent the intrinsic rate of increase *r*, the net reproductive rate *R*_*0*_, the mean generation times *T* and the finite rate of increase *λ*. In our study, the main life table parameters researched were the population parameters.

The intrinsic rate of increase (*r*) of the treatments was significantly lower than that of the controls. The intrinsic rate of increase is a key demographic parameter that has been used to summarize the reproductive potential of an animal population in relation to its capacity to increase^29^. Southwood^37^ stated that the intrinsic rate of increase is the most useful life table parameter for comparing the population growth potential under specific climatic and food conditions. Therefore, the negative effect of *R. insecticola* on the potential growth of *S. avenae* clones is intriguing. This notion was confirmed by the mean generation times (*T*) of the treatments, which were shorter than that of the controls. Oliver *et al*.^25^ reported that *H. defensa* shortened the mean generation time (*T*) of *A. pisum*. Although *R. insecticola* might decelerate the potential growth of *S. avenae* populations, the finite rates of increase (*λ*) were all over 1 between the treatments and the controls, indicating that the studied *S. avenae* population increased continuously. The net reproductive rates (*R*_0_ > 1) between the treatments and the controls also confirmed this observation. However, the finite rates of increase (*λ*) of the treatments with the secondary symbionts were remarkably less than that of the controls, suggesting that the daily increase of *S. avenae* clones in the treatment groups was significantly lower than that of the controls. This was confirmed by the birth rates of the treatment groups, which were significantly lower than that of the controls. In conclusion, *R. insecticola* may reduce the potential growth of *S. avenae* clones, and this is consistent with the findings of Wang *et al*.^27^, suggesting a possibility of a cost action to harbor this secondary endosymbiont. It was expected that the secondary endosymbiont would play a role against the infection of fungal pathogens^17^.

Compared to the controls, we could conclude that *R. insecticola* increased the time of pre-adult duration. The results are consistent with the report by Wang *et al*.^27^. In addition, there was the positive effect of the secondary endosymbionts on the longevity of *S. avenae* clones compared to that of the Linyi control. However, there was no effect of the secondary endosymbionts on the clones of Neixiang-1, Neixiang-2 and Neixiang-NA, which may be explained by the difference of geographic populations.

The intrinsic rates of increase (*r*), finite rates of increase (*λ*), mean generation time (*T*) and birth rate demonstrated that *R. insecticola* infection had a negative effect on the natural development of *S. avenae* clones, and the impact levels differed among different genetic backgrounds. The difference between genotypes might be due to variations in host nutrients. For example, the effect of nitrogen content and protein quality might affect the fecundity of herbivores^38^,^39^,^40^,^41^. Therefore, to accurately and systematically assess the ecological effect of all endosymbionts on aphids, (1) the endosymbiont treatment and genotype interaction should be noted in future studies, (2) all endosymbionts (i.e., primary and secondary), such as *S. symbiotica, H. defense and Rickettsia*, need to be examined individually, and (3) more donor and recipient clones or species could be used, as only 2 aphid clones were used as donors and 2 aphid clones were recipients in the current study. Such efforts would provide us with a comprehensive perspective that would aid our understanding of the complex symbiotic relationships between aphids and bacterial endosymbionts.

In conclusion, the findings of this study indicate that the interaction effects between *R. insecticola* and the genotypes of *S. avenae* clones did not show any changes in fitness traits, although changes were observed with the net reproductive rate (*R*_0_), pre-adult duration and total longevity. In addition, the significant reduction of fitness traits (e.g., *r, λ* and birth rate) and the clear increase of the mean generation times (*T*) indicated that *R. insecticola* might have a negative effect on the potential population growth of *S. avenae*.

## Materials and Methods

### Aphid sources

Aphid clones (considered genotypes in this paper) used in this study were established from individuals collected from winter wheat plants on private farm land, with the permission of the owners. Clones without *R. insecticola* were collected in Neixiang (33.03°N, 111.50°E), Henan Province and in Linyi (35.05°N, 118.35°E), Shandong Province in May 2014. Collected clones were genotyped at four microsatellite loci (S91, S24, S30, and Sm10) as described in the Supplementary Information (Tables S1 and S2). *R. insecticola* donor clones were collected in April 2014 in two quadrats, at a distance of 300 m from each other, in Yangpingguan (34.8°N, 105.6°E), Shaanxi. They were named Yangpingguan 1 and Yangpingguan 2. Each clone was derived from a single parthenogenetic *S. avenae* female, and the endosymbionts of the four clones were detected. The primers are shown in the Supplementary Information (Table S3). Clones were laboratory-reared at 20 °C under a 12 h light: 12 h dark cycle in separate cubical cages (side length = 25 cm) on fresh wheat (*Triticum aestivum* L.) seedlings (variety ‘1376’) that were changed at ten-day intervals^32^.

### Transfer of *R. insecticola* and further detection

The micro-injection technique advocated by Chen & Purcell^42^ was used to inoculate *R. insecticola* into either 2^nd^ or 3^rd^ instar *S. avenae* individuals. To do this, the Yangpingguan clones 1 and 2 (from Shaanxi) that hosted *R. insecticola* were chosen as the donor clones. Body fluids from the Yangpingguan clones 1 and 2 were transferred into the Linyi (from Shandong) and the Neixiang clones (from Henan), respectively, resulting in the creation of four new clones (Linyi-1 and Neixiang-1 from Yangpingguan clone 1 donor, Linyi-2 and Neixiang-2 from Yangpingguan clone 2 donor). Therefore, the Linyi-1 and Linyi-2 clones and the Neixiang-1 and Neixiang-2 clones have the same genetic background as the Linyi (Shandong) and the Neixiang clones (Shandong), respectively. The surviving aphids (approximately 20 individuals for clones Linyi-1, Linyi-2, Neixiang-1, and Neixiang-2) after the injection were transferred to fresh wheat plants (one aphid per plant). Twelve hours later, the dead aphids were discarded, and the remaining surviving aphids were reared till they became adults and produce nymphs. Next, one single offspring individual from each clone that tested “positive” was subjected to PCR using PCR primers described in Sandström *et al*.^9^, *Taq* DNA polymerase mix, and the following cycle parameters: 94 °C for 7 min and 35 cycles of 94 °C for 30 s, 57 °C for 45 s and 72 °C for 30 s. The resulting PCR products were separated in a 1% agarose gel and visualized under UV after gel staining with ethidium bromide for the presence of *R. insecticola* and were then placed on *T. aestivum* and allowed to reproduce for several days. At this time, adults were removed and 5 individuals were tested again for the presence of *R. insecticola*. One positive clone was then used to conduct the following study. To ensure that *R. insecticola* densities in *S. avenae* produced clones approached equilibrium, the original data of the life table were collected after a minimum of 10 generations^5^ and statistically analyzed.

### Life table study

Linyi-1 and Linyi-2 clones were compared with the Linyi clone control as they all shared a common genetic background. This clone was named Linyi-NA for convenience. Similarly, Neixiang-1 and Neixiang-2 clones were compared with the Neixiang clone control, and the control was named Neixiang-NA for convenience. A preliminary experiment showed that the control clone (no injection) and the negative control (injection of water) showed no difference in the growth and development of the studied *S. avenae* clones (unpublished data). Experiments were performed using three groups for each individual clone, with a total of 30 aphids in each group. Newly emerged 1^st^ instar nymphs were individually transferred within 24 h to a 9-cm Petri dish containing a fresh wheat leaf blade to start the experiment. The collection of life table data was initiated approximately 24 h later. Each aphid was observed daily at the same time until all individuals died. All offspring were removed daily, and the wheat leaves were replaced every 2 days. Molting and mortality events, as well as the number of produced nymphs, were recorded.

### Life table analysis

The developmental time and daily fecundity of all individuals were analyzed according to an age-stage, two-sex life table^43^,^44^ using the program TWOSEXMS Chart^45^. The intrinsic rate of increase (*r*) was estimated using an interactive bisection method and the following Euler–Lotka equation (equation 1), with the age indexed from 0^46^:


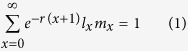


The net reproductive rate (*R*_0_) was calculated as follows (equation 2):


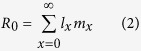


The finite rate of increase *λ* (equation 3) and the mean generation time *T* (equation 4) were calculated as follows:


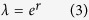



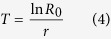


### Population projection

Based on the results of the age-stage, two-sex life table, a TIMING-MS Chart was used to predict the population growth of the studied *S. avenae* clones. For this prediction, we assumed unlimited growth with an initial population of 10 pairs of newly emerged *S. avenae* nymphs^29^,^47^,^48^.

### Experimental design and data analysis

A 2 × 3 factorial randomized in complete blocks with 30 replications was deployed to test *R. insecticola* on *Sitobion avenae* clones. All data were analyzed using IBM SPSS version 20. The differences in life history traits produced from the life table were separately subjected to a linear two-way nested analysis of variance (ANOVA) to analyze the effects of ‘*R. insecticola*’ and clone (i.e., ‘Linyi’ and ‘Neixiang’) nested in ‘*R. insecticola*’. We used Tukey’s procedure^49^ to compare the differences among treatments, following the description of Sokal & Rohlf^50^. All mean separation tests were performed at *P* < 0.05. Mean values (±standard errors of the mean) were calculated and used in the above analyses. If needed, data were transformed to meet the required assumptions of normality and homoscedasticity. Graphs were generated using SigmaPlot 12.5.

## Additional Information

**How to cite this article:** Luo, C. *et al*. Ecological impact of a secondary bacterial symbiont on the clones of *Sitobion avenae* (Fabricius) (Hemiptera: Aphididae). *Sci. Rep.* **7**, 40754; doi: 10.1038/srep40754 (2017).

**Publisher's note:** Springer Nature remains neutral with regard to jurisdictional claims in published maps and institutional affiliations.

## Supplementary Material

Supplementary Information

## Figures and Tables

**Table 1:** Estimates of variance components for population parameters (i.e., the intrinsic rate of increase *r*, the mean generation times *T*, the finite rate of increase *λ* and the net reproductive rate *R*_*0*_) of *Sitobion avenae* clones showing the main effects of *Regiella insecticola* and clone (i.e., ‘Linyi’ and ‘Neixiang’) nested in the *Regiella insecticola*.

	Intrinsic rate of increase *r*					Mean generation times *T*					Finite rate of increase *λ*					Net reproductive rate *R*_*0*_				
	SS	df	MS	*F*	*P*	SS	df	MS	*F*	*P*	SS	df	MS	*F*	*P*	SS	df	MS	*F*	*P*
*R. insecticola*	0.010	2	0.005	38.448	**<0.001**	15.321	2	7.661	22.527	**<0.001**	0.018	2	0.009	39.547	**<0.001**	109.385	2	54.693	9.246	**0.004**
Clone (*R. insecticola*)	0.001	3	<0.001	2.727	0.091	1.656	3	0.552	1.623	0.236	0.002	3	0.001	2.730	0.090	212.312	3	70.771	11.965	**0.001**
Error	0.002	12	<0.001	—	—	4.081	12	0.340	—	—	0.003	12	<0.001	—	—	70.980	12	5.915	—	—

A linear two-way nested analysis of variance (ANOVA) was performed. Bold text indicates significant effects.

**Table 2:** The effects of *R. insecticola* on the intrinsic rate of increase *r*, the net reproductive rate *R*_*0*_, the mean generation times *T*, and the finite rate of increase *λ* of the studied *S. avenae* clones (Linyi-1, Linyi-2, Neixiang-1, Neixiang-2, Linyi-NA and Neixiang-NA) (Mean ± SE).

Clones	Genotype	*R. insecticola donor*	Population parameter (Mean ± SE)			
			Intrinsic rate of increase *r* (d^−1^)	Net reproductive rate *R*_*0*_(offspring/aphid)	Mean generation times *T* (d)	Finite rate of increase *λ* (d^−1^)
Linyi-1	Linyi	Yangpingguan 1	0.24 ± 0.003 b	38.04 ± 1.333 a	14.97 ± 0.287 a	1.28 ± 0.004 b
Linyi-2	Linyi	Yangpingguan 2	0.28 ± 0.010 b	42.50 ± 0.896 a	13.64 ± 0.472 a	1.32 ± 0.013 b
Linyi-NA	Linyi	control	0.30 ± 0.003 a	39.14 ± 2.622 a	12.06 ± 0.260 b	1.36 ± 0.004 a
Neixiang-1	Neixiang	Yangpingguan 1	0.26 ± 0.009 b	38.20 ± 0.636 a	13.91 ± 0.430 a	1.30 ± 0.012 b
Neixiang-2	Neixiang	Yangpingguan 2	0.26 ± 0.007 b	34.43 ± 0.667 a	13.66 ± 0.340 a	1.30 ± 0.009 b
Neixiang-NA	Neixiang	control	0.31 ± 0.000 a	47.89 ± 1.234 a	12.33 ± 0.084 b	1.37 ± 0.000 a

Different letters (a, b) within a column indicate statistically significant differences (*P* < 0.05; Tukey’s test was used for mean separation).

**Table 3:** Estimates of variance components for life history traits (i.e., the birth rate, pre-adult duration and total longevity) of *Sitobion avenae* clones showing the main effects of *Regiella insecticola* and clone (i.e., ‘Linyi’ and ‘Neixiang’) nested in the *Regiella insecticola*.

	Birth rate					Pre-adult duration					Total longevity				
	SS	df	MS	F	P	SS	df	MS	F	P	SS	df	MS	F	P
*R. insecticola*	0.018	2	0.009	40.872	**<0.001**	4.910	2	2.455	37.663	**<0.001**	29.211	2	14.605	13.200	**0.001**
Clone (*R. insecticola*)	0.002	3	0.001	2.721	0.091	0.974	3	0.325	4.980	**0.018**	26.162	3	8.721	7.881	**0.004**
Error	0.003	12	<0.001	—	—	0.782	12	0.065	—	—	13.278	12	1.106	—	—

A linear two-way nested analysis of variance (ANOVA) was performed. Bold text indicates significant effects.

**Table 4:** The effects of *R. insecticola* on the birth rate, pre-adult duration and total longevity of the studied *S. avenae* clones (Linyi-1, Linyi-2, Neixiang-1, Neixiang-2, Linyi-NA and F Neixiang-NA Mean ± SE).

Clones	Genotype	*R. insecticola donor*	Life-history traits (Mean ± SE)		
			Birth rate	Pre-adult duration (d)	longevity (d)
Linyi-1	Linyi	Yangpingguan 1	0.28 ± 0.004 b	8.32 ± 0.062 a	27.34 ± 0.484 a
Linyi-2	Linyi	Yangpingguan 2	0.32 ± 0.013 b	7.51 ± 0.156 a	27.62 ± 0.624 a
Linyi-NA	Linyi	control	0.36 ± 0.004 a	7.03 ± 0.126 b	22.29 ± 0.756 b
Neixiang-1	Neixiang	Yangpingguan 1	0.30 ± 0.011 b	7.80 ± 0.102 a	26.31 ± 0.791 a
Neixiang-2	Neixiang	Yangpingguan 2	0.30 ± 0.009 b	7.97 ± 0.259 a	25.41 ± 0.423 a
Neixiang-NA	Neixiang	control	0.37 ± 0.001 a	6.62 ± 0.950 b	25.68 ± 0.453 a
