# Supplementary material for: Ecological impact of a secondary bacterial symbiont on the clones of Sitobion avenae (Fabricius) (Hemiptera: Aphididae)
Source: Sci Rep. 2017 Jan 17;7:40754. doi: 10.1038/srep40754 (PMC5240142; doi:10.1038/srep40754)
Supplement: Supplementary Information [file srep40754-s1.pdf]

**Title** Ecological impact of a secondary bacterial symbiont on the clones of *Sitobion avenae*(Fabricius) (Hemiptera: Aphididae)

Chen Luo[1,2, a], Kun Luo[1,a], Lingqin Meng[1], Bin Wan[2], Huiyan Zhao[1] & Zuqing Hu[1,*]

[1]State Key Laboratory of Crop Stress Biology for Arid Areas, College of Plant Protection, Northwest A&F University, Yangling, Shaanxi Province 712100, China.

[2]INRA (French National Institute for Agricultural Research), Univ. Nice Sophia Antipolis, CNRS, UMR 1355-7254 Institut Sophia Agrobiotech, 06903, Sophia Antipolis, France.

[a]These authors contributed equally to this work.

*Corresponding author

E-mail: huzuqing@nwsuaf.edu.cn (ZH)

## Supplementary information

**Table S1.** Microsatellite markers of distinguishing the *Sitobion avenae* clones used in this study. For all reactions, the PCR cycling conditions were in[1].

| **Marker** | **Forward Primer (5'-3')** | **Reverse Primer (5'-3')** | **Ref.** |
|---|---|---|---|
| S19 | GCGCATTGTGTAGCGAGC | CAAACATGTTATGTCACAATAC | 1 |
| S24 | CCCGACCCCGTCCATTCAAA | CCTCCACCACTACTTTCACTCC | 1 |
| S30 | CCGACATAAAACACACCCAG | GTTTTGCCTCCTCCCCTC | 1 |
| Sm10 | TCTGCTGCATTACTGTTGGC | TCGTCTACTTCGCCGTCA | 2 |

**Table S2.** Microsatellite profiles of the two aphid clones used in the study.

| **Clone** | **S19** | **S24** | **S30** | **Sm10** |
|---|---|---|---|---|
| Linyi (35.05 °N, 118.35 °E) | 227/230 | 166/175 | 159/172 | 164/164 |
| Neixiang (33.03 °N, 111.50 °E) | 207/223 | 166/183 | 171/171 | 153/166 |

**Table S3.** Endosymbiotic bacteria examined in this study.

| Target symbiont | Target gene | Primer name | Primer Sequence(5'-3') | Tm | Product size (kb) | Refs |
|---|---|---|---|---|---|---|
| *R. insecticola* | 16SrDNA | U99F | ATCGGGGAGTAGCTTGCTAC | 57 °C | 1.3 | 3 |
| | | 1507R | TACCTTGTTACGACTTCACCCCAG | | | |
| *H. defensa* | 16SrDNA | PABSF | AGCACAGTTTACTGAGTTCA | 59 °C | 1.3 | 4 |
| | | 16SB1 | TACGGYTACCTTGTTACGACTT | | | 5 |
| *S. symbiotica* | 16SrDNA | 16SA1 | AGAGTTTGATCMTGGCTCAG | 59 °C | 0.48 | 5 |
| | | PASScmp | GCAATGTCTTATTAACACAT | | | 6 |
| *Spiroplasma* | 16SrDNA | 16saiF | AGAGTTTGATCMTGGCTCAG | 61 °C | 0.35 | 5 |
| | | TKSSspR | TAGCCGTGGCTTTCTGGTAA | | | 6 |
| *Arsenophonus* | 23SrDNA | Ars23sF | CGTTTGATGAATTCATAGTCAAA | 57 °C | 0.55 | 7 |
| | | Ars23sR | GGTCCTCCAGTTAGTGTTACCCAAC | | | |
| *Rickettsia* | 16SrDNA | 16saiF | AGAGTTTGATCMTGGCTCAG | 60 °C | 0.20 | 5 |
| | | Rick16sR | CATCCATCAGCGATAAATCTTTC | | | 6 |
| *Rickettsiella* | 16SrDNA | Ric-470R | TGGGTACCGTCACAGTAATCGA | 60 °C | 0.30 | 8 |
| | | P136F | GGGCCTTGCGCTCTAGGT | | | |
| PAXS | 16SrDNA | PAXS F | AGTTTGATCATGGCTCAGATTG | 60 °C | 0.50 | 8 |
| | | PAXS R | GCAACACTCTTTGCATTGCT | | | |
| *Buchnera* | 16SrDNA | ArsCAIF | GCCTGATGCAGCCATGCCGCGTGTATG | 55 °C | 0.50 | 9 |
| | | ArsCAIR | GTCATCCCCACCTTCC | | | |

## References


1. Wilson, A.C.C. *et al.* Cross-species amplification of microsatellite loci in aphids: assessment and application. *Mol. Ecol. Notes.* **4**, 104-109 (2004).
2. Simon, J.C. *et al.* Reproductive mode and population genetic structure of the cereal aphid *Sitobion avenae* studied using phenotypic and microsatellite markers. *Mol. Ecol.* **8**, 531-545 (1999).
3. Sandström, J.P., Russell, J.A., White, J.P. & Moran, N.A. Independent origins and horizontal transfer of bacterial symbionts of aphids. *Mol. Ecol.* **10,** 217-228 (2001).
4. Darby, A.C., Birkle, L.M., Turner, S.L. & Douglas, A.E. An aphid-borne bacterium allied to the secondary symbionts of whitefly. *FEMS. Microbiol. Ecol.* **36,** 43-50 (2001).
5. Fukatsu, T. & Nikoh, N. Two intracellular symbiotic bacteria from the mulberry psyllid *Anomoneura mori* (Insecta, Homoptera). *Appl. Environ. Microb.* **64,** 3599-3606 (1998).
6. Fukatsu, T., Tsuchida, T., Nikoh, N. & Koga, R. *Spiroplasma* symbiont of the pea aphid, *Acyrthosiphon pisum* (Insecta: Homoptera). *Appl. Environ. Microb.* **67,** 1284-1291 (2001).
7. Thao, M.L.L. & Baumann, P. Evidence for multiple acquisition of *Arsenophonus* by whitefly species (Sternorrhyncha : Aleyrodidae). *Curr. Microbiol.* **48,** 140-144 (2004).
8. Peccoud, J. *et al.* Inheritance patterns of secondary symbionts during sexual reproduction of pea aphid biotypes. *Insect. Sci.* **21,** 291-300 (2014).
9. Dale, C. & Moran, N.A. Molecular interactions between bacterial symbionts and their hosts. *Cell.* **126,** 453-465 (2006).
